# NLK functions to maintain proliferation and stemness of NSCLC and is a target of metformin

**DOI:** 10.1186/s13045-015-0203-8

**Published:** 2015-10-26

**Authors:** Dong Suwei, Zeng Liang, Liu Zhimin, Li Ruilei, Zou Yingying, Li Zhen, Ge Chunlei, Lai Zhangchao, Xue Yuanbo, Yang Jinyan, Li Gaofeng, Song Xin

**Affiliations:** Cancer Research Institute of Southern Medical University, Guangzhou, Guangdong People’s Republic of China; Department of Cancer Biotherapy Center, The Third Affiliated Hospital of Kunming Medical University (Tumor Hospital of Yunnan Province), Kunming, Yunnan People’s Republic of China; Department of Pathology, Hunan Tumor Hospital, Changsha, Hunan People’s Republic of China; Department of Pathology and Pathophysiology, Kunming Medical University, Kunming, Yunnan People’s Republic of China; Department of Thoracic Surgery, The Third Affiliated Hospital of Kunming Medical University (Tumor Hospital of Yunnan Province), Kunming, Yunnan People’s Republic of China

**Keywords:** NLK, NSCLC, Proliferation, Metformin, Stemness

## Abstract

**Objective:**

Nemo-like kinase (NLK) is an evolutionarily conserved serine/threonine kinase that regulates the activity of a wide range of signal transduction pathways. Metformin, an oral antidiabetic drug, is used for cancer prevention. However, the significance and underlying mechanism of NLK and metformin in oncogenesis has not been fully elucidated. Here, we investigate a novel role of NLK and metformin in human non-small cell lung cancer (NSCLC).

**Materials and methods:**

NLK expression was analyzed in 121 NSCLCs and 92 normal lung tissue samples from benign pulmonary disease. Lentivirus vectors with NLK-shRNA were used to examine the effect of NLK on cell proliferation and tumorigenesis in vitro. Then, tumor xenograft mouse models revealed that NLK knockdown cells had a reduced ability for tumor formation compared with the control group in vivo. Multiple cell cycle regulator expression patterns induced by NLK silencing were examined by western blots in A549 cells. We also employed metformin to study its anti-cancer effects and mechanisms. Cancer stem cell property was checked by tumor sphere formation and markers including CD133, Nanog, c-Myc, and TLF4.

**Results:**

Immunohistochemical (IHC) analysis revealed that NLK expression was up-regulated in NSCLC cases (*p* < 0.001) and correlated with tumor T stage (*p* < 0.05). Silencing of NLK suppressed cell proliferation and tumorigenicity significantly in vitro and in vivo, which might be modulated by JUN family proteins. Furthermore, metformin selectively inhibits NLK expression and proliferation in NSCLC cells, but not immortalized noncancerous lung bronchial epithelial cells. In addition, both NLK knockdown and metformin treatment reduced the tumor sphere formation capacity and percentage of CD133+ cells. Accordingly, the expression level of stem cell markers (Nanog, c-Myc, and TLF4) were decreased significantly.

**Conclusion:**

NLK is critical for cancer cell cycle progression, and tumorigenesis in NSCLC, NLK knockdown, and metformin treatment inhibit cancer cell proliferation and stemness. Metformin inhibits NLK expression and might be a potential treatment strategy for NSCLC.

## Introduction

Lung cancer is the most common cancer worldwide, and is the leading cause of cancer mortality killing 1.4 million people annually [1]. Non-small cell lung cancer (NSCLC) comprises approximately 80 % of all lung cancer cases. Despite recent developments in treatment strategies for NSCLC, the prognosis remains unsatisfactory, with an overall 5-year survival rate of only 10–15 % [2]. Therefore, it is critical to identify new proteins responsible for NSCLC development and progression to provide targets for the successful treatment of lung cancer.

Serine/threonine kinases (STKs) play a key role in cellular signaling through their ability to phosphorylate transcription factors, cell cycle regulators, and a wide array of other cytoplasmic and nuclear effectors [3]. Their dysregulation is frequently linked to disease and targeted therapeutic approaches have been aimed at identifying drugs with the ability to modulate their function [4, 5].

Nemo-like kinase (NLK) is an evolutionarily conserved serine/threonine kinase that belongs to the proline directed protein kinase superfamily which consists of mitogen-activated protein kinases (MAPKs) and cyclin-dependent protein kinases (CDKs) [6]. Previous studies suggested that it is involved in multiple developmental processes in Drosophila [7–9], and recent work shows that NLK can regulate the activity of a wide range of transcription factors either directly or indirectly [10, 11]. Aberrant expression of NLK has been reported in human cancer including colon [12], hepatocellular [13], and prostate carcinomas [14]. However, at present, the significance of NLK expression in oncogenesis is still not fully understood.

Metformin, an oral antidiabetic drug, can effectively reduce morbidity and mortality of malignant tumor patients with type 2 diabetes [15]. It is also widely used in the prevention of breast cancer, prostate cancer, and other tumors [16, 17]. However, the role of metformin in NSCLC has not been carefully characterized. It is suggested that metformin plays an anti-tumor effect mainly by acting on serine/threonine kinases like AMPK and mTOR [18]. We hypothesized that NLK maybe a downstream effector of metformin.

In this study, we demonstrate that the expression of NLK is up-regulated in NSCLC compared with benign lung tissue. Knockdown of NLK in both A549 and SK-MES-1 cells reduced their proliferation in vitro, as well as tumor growth in vivo. As underlying mechanisms, cell cycle regulators are modulated by NLK suppression and lead to G1 phase arrest. The effects from NLK silencing are recapitulated by metformin treatment, which also reduces NLK expression in NSCLC cells. Furthermore, both knockdown of NLK and metformin treatment significantly reduced cancer stem cell stemness. Taken together, our findings indicate that NLK plays a crucial role in proliferation and tumorigenesis of NSCLCs, and suggest that NLK could be a potential therapeutic target in NSCLC treatment. Meanwhile, metformin can be a potential drug for NSCLC, specifically through inhibition of cancer cell proliferation, down-regulation of NLK, and reduction of the cancer stem cell population.

## Materials and methods

### Clinical samples

Two-hundred thirteen formalin-fixed, paraffin-embedded tissue specimens were obtained from the Department of Pathology at the Third Affiliated Hospital of Kunming Medical University (Tumor Hospital of Yunnan Province). The specimens consisted of 121 primary NSCLC and 92 lung tissue samples from benign pulmonary diseases (including 45 samples of pneumonia, 42 inflammatory pseudotumors, 4 bullas, and 1 sillicosis). For the NSCLC cases, there were 60 adenocarcinomas and 61 squamous cell carcinomas, respectively, including 85 males and 36 females, with ages ranging from 33 to 73 years (mean age, 55.5 years). All patients underwent primary tumor resection plus lymph node dissection. Patients with a diagnosis of relapse and who had received preoperative radiation, chemotherapy, or biotherapy were excluded from the study to avoid any changes in tumor marker determination resulting from treatment. Patients diagnosed with multiple primary cancers in other organs or tissues were also excluded. The study was approved by the ethics committee of the Third Affiliated Hospital of Kunming Medical University, and all patients gave written informed consent and authorization for use of biological specimens. Demographic and clinical data were obtained from the patients’ medical records.

### Cell lines

Lung cancer cell lines used in this study were NCI-H522, NCI-H2342, NCI-H2405, NCI-A549 and SPC-A-1 (human lung adenocarcinoma cell lines), SW900, NCI-H1869 and SK-MES-1 (human lung squamous cancer cell lines), NCI-H661, NCI-H1299 and NCI-H1581 (human large cell lung cancer cell lines), and BEAS-2B (human lung epithelial cell line). The SPC-A-1 cell line was purchased from Chinese Academy of Sciences Cell Bank, and others were obtained from ATCC Bioresource Center. All cancer cell lines were grown in RPMI 1640 supplemented with 10 % FCS and ampicillin/streptomycin. BEAS-2B was cultured in BEGM.

### Pathology

**Fig. 1:**
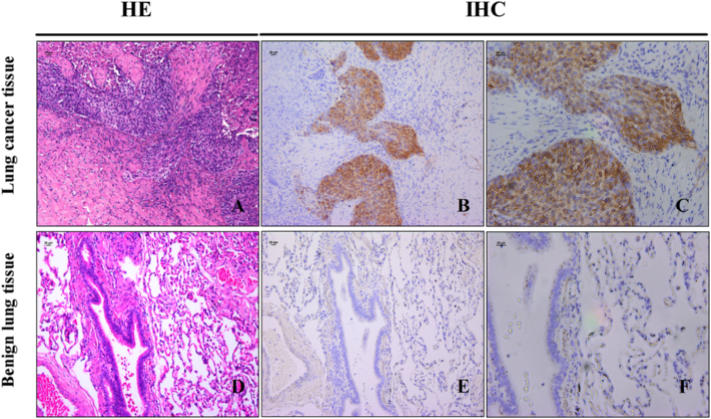
NLK expression is up-regulated in NSCLC tissues. **a** Hematoxylin and eosin staining of lung squamous cell carcinoma tissue. **b**, **c** Immunohistochemical staining for NLK, showing positive immunostaining in NSCLC tissue. Pathologic tissue section no. 3257, *scale bar*, 20 μm. **d** Hematoxylin and eosin staining of benign lung tissue. **e**, **f** Immunohistochemical staining for NLK, showing negative immunostaining in benign lung tissue. Pathologic tissue section no. 1828, *scale bar*, 20 μm

A routine histological examination was performed with hematoxylin-eosin staining (Fig. 1a, b) and reviewed independently by three pathologists. Benign lung tissues were collected from a normal part of benign pulmonary diseases identified by pathologists. All carcinomas were classified in accordance with the 7th edition of the AJCC cancer staging system [19].

### Immunohistochemistry (IHC) analysis

Samples were processed for immunohistochemical analysis to determine NLK expression levels and distribution patterns. 4-μm sections of paraffin-embedded tissues were mounted on charged glass slides and baked at 60 °C for 2 h. The slides were allowed to cool to room temperature, deparaffinized in xylene, and rehydrated in a graded alcohol series. Sections were microwave-treated for 10 min in a citrate buffer (pH 6.0) for antigen retrieval, and endogenous peroxidase activity was blocked by incubation in 0.3 % hydrogen peroxide for 10 min. Rabbit polyclonal antibodies (HPA018192, Sigma, USA) were used to detect NLK protein at a 1:25 dilution in phosphate-buffered saline (PBS). After two washes in PBS, slides were incubated with ABC (Vector Laboratories, Burlingame, CA, USA), washed, overlaid with 3-30-diaminobenzidine (DAB, Dako Corporation, Carpinteria, CA, USA), and counterstained with hematoxylin. Human hepatocellular carcinoma tissues were used as positive controls, and negative controls were obtained by replacing the primary antibody with non-immunized serum. Sections were scored semi-quantitatively for the extent of immunoreaction as follows: 0, 0 % immunoreactive cells; 1, <5 % cells; 2, 5–50 % immunoreactive cells; and 3, >50 % immunoreactive cells. In addition, the intensity of staining was scored semi-quantitatively as 0, negative; 1, weak; 2, intermediate; and 3, strong. The final immunoreaction score was defined as the sum of both parameters (extent and intensity), and samples were grouped according to the summed score as negative (0), weak staining (1–2), moderate staining (3) and strong staining (4–6) [20]. Final immunoreaction scores >0 were defined as positive. All slides were evaluated independently for protein expression by three separate observers, and slides with an incongruent grading were scrutinized a second time and a consensus was reached.

### Short hairpin RNA (shRNA)

To stably suppress NLK expression, we constructed two shRNA against NLK. sh-NLK1: 5’-CGGATAGACCTACCGGATATG-3’, sh-NLK2: 5’-GAATATCCGCTAAGGATGC-3’ with scramble shRNA: 5’-TTCTCCGAACGTGTCACGT-3’). The double-stranded shRNA was inserted into the pFU-GW-RNAi plasmid containing GFP gene and then linearized with Hpa I (GTTAAC) and Xho I (CTCGAG), termed as pFU-GW-NLK-shRNA. To produce lentiviruses, 293 T cells were transiently cotransfected with pFU-GW-NLK-shRNA together with pHelper 1.0 vector and pHelper 2.0 vector by Lipofectamine 2000 (11668-027, invitrogen, USA). Packaged viruses were harvested from supernatant of the 293 T transfected cells.

### Cell infection

Lentiviral infections were carried out as follows: briefly, when the cells reached 30–50 % confluence NLK-shRNA or the scramble RNA were added into the medium at a multiplicity of infection (MOI) of 30 PFU/cell for A549 and 15 PFU/cell for SK-MES-1, and cells were incubated for 10 h with Polybrene (5 μg/ml) at 37 °C in 5 % CO_2_. Infection efficiency was determined by monitoring the GFP signal (the transfection efficiency of cells were more than 90 %). A parallel culture of cells without any treatment served as the negative control.

### Construction of the plasmid vector and DNA transfection

Based on the NLK sequence available on the National Center for Biotechnology Information database, the full-length NLK sequence was synthesized and subcloned into pEGFP-N1. The clones were confirmed by restriction analysis and DNA sequencing of both strands (Invitrogen, Shanghai, China). The empty vector or the pEGFP-N1-NLK vector was transfected into cells using Lipofectamine 2000 (Invitrogen, Carlsbad, CA, USA), according to the manufacturer’s instructions. The empty pEGFP-N1 vector was used as the negative control.

### Immunoblot

Cells were harvested 72 h after transfection and lysed in RIPA buffer (89900, Pierce, USA). The lysates were centrifuged at 14,000 rpm for 20 min at 4 °C, and the supernatants collected. Protein concentration was measured using the BCA assay (23227, Thermo, USA). For each sample, 50 μg of protein lysate was loaded per well. Samples were electrophoresed on 10 % SDS-PAGE gels and transferred onto polyvinylidene fluoride (PVDF) membranes (ISEQ00010, Millipore, USA) by electro blotting. Membranes were pretreated with 5 % nonfat dry milk in TBS-T for 2 h, followed by incubation with primary antibody for 16 h. All the following primary antibodies used were diluted at 1:1000 and purchased from Abcam, USA: NLK (ab26050), Cyclin A2 (ab38), Cyclin D1 (ab6152), Cyclin D2 (ab3085), Cyclin D3 ab28283), Cyclin E1 (ab3927), Cyclin E2 (ab32103), CDK2 (ab2363), CDK4 (ab108357), CDK6 (ab3126), p21 (ab7960), p27 (ab32034), c-Jun (ab32137), c-Jun pSer63 (ab32385), c-Jun pSer73 (ab30620), JunB (ab128878), JunB pSer259 (ab30628),JunD (ab28837), JunD pSer255 (ab2883). The membranes were washed three times with TBS-T. Then, the membranes were incubated with horseradish peroxidase (HRP)-labeled secondary antibody (1:10, 000, #7076, Cell Signaling, USA) for 1 h before detection by ECL (RPN2135, GE healthcare, UK). α-tubulin was used as an internal loading control (1:1000, #2125, Cell Signaling, USA).

### Cell counting

Briefly, for cell counts, cells were seeded in 24-well plates at a density of 1 × 10^4^ cells per well and three duplicate wells were set up for each group. Cells were harvested with trypsin/EDTA every 24 h for 7 days, and counted in an electronic cell counter (Coulter Z1, Beckman-coulter, Fullerton, CA, USA). Growth curves were generated to assess proliferation. All experiments were done in triplicate.

### MTS assay

Cell proliferation was also quantified by performing the 3-(4, 5-dimethylthiazol -2-yl)-5-(3-carboxymethoxyphenyl)-2-(4-sulfophenyl)-2H-tetrazolium (MTS) assay. Briefly, 4 × 10^3^ cells were plated per well in 96-well culture plates in 150 μl of medium, and six parallel wells were assigned to each group, as well as a negative control (without cells). Over a 7-day period, every 24 h, 30 μl of MTS substrate was added to each well, and then incubated for 2 h in the dark. The absorbance at 490 nm was measured for each sample using a plate reader (BMG Labtech, Offenburg, Germany). The concentrations required to inhibit growth by 50 % (IC50) were calculated from survival curves using the Bliss method [21]. All experiments were performed three times independently.

### Flow cytometric analysis

Flow cytometric analysis of cell cycle phase distribution and CD133+ cell percentage were carried out utilizing DNA content or CD133-PE antibody (130-098-826, Meltenyi Biotec, Germany). Cells were harvested by trypsinization and washed three times with cold PBS by centrifugation at 300 g for 5 min. For cell cycle analysis, cells were fixed in pre-chilled 70 % ethanol overnight at 4 °C. The fixed cells were collected, washed twice with PBS, suspended in PBS containing 10 μg/ml propidium iodide (Sigma, USA) and 100 μg/ml RNase A (Roche, Switzerland), then incubated at 37 °C for at least 30 min in the dark to eliminate intracellular RNA. For CD133+ cell analysis, resuspended cells were incubated with CD133-PE or IgG1-PE antibody (130-093-193, Meltenyi Biotec, Germany) at 4 °C for 15 min, followed by centrifugation and resuspension in PBS. Analysis was done on a FACS Aria system (BD Immunocytometry Systems, San Jose, CA, USA) and analyzed by Cell Quest software (Becton Dickinson Ltd). All of the samples were assayed three times.

### Nude mice xenograft assay

Athymic female BALB/c nude mice (4–5 weeks of age, 20–25 g) were obtained from Beijing Vital River Laboratory Animal, Inc. (Beijing, China). All experimental procedures were approved by the Institutional Animal Care and Use Committee of Kunming Medical University. Mice were randomly divided into 3 groups (*n* = 5/group). Animals had free access to water and standard food, and their health was monitored daily. The mice (5 per cage) were housed under specific pathogen free conditions with controlled light (12 h light, 12 h darkness) and temperature (21 °C). Since Matrigel can act as a growth-promoting factor after tumor cell injection [22, 23], we generated mouse tumor models by subcutaneously inoculating A549, A549-scramble or A549-shRNA cells mixed with Matrigel as previous described [24]. Briefly, 8 × 10^6^ cells were suspended in 50 μl PBS and mixed with 50 μl Matrigel (354234,BD,USA) in a 1-ml syringe, allowed to warm at room temperature for 10 min with gentle mixing, and then injected into the right flank of the nude mice. The length (a) and width (b) of the xenograft tumors were measured every 3 days with a Vernier caliper. Tumor volumes (cm^3^) were calculated by the following formula: volume = ab2/2. Mice were sacrificed 7 weeks after inoculation, and tumors were excised and weighed.

### Tumor sphere formation assay

Sphere formation assay was conducted as previously described [25]. Briefly, A549 cells were separated into four groups, untreated, infected with scramble RNA, sh-NLK1, and sh-NLK2 with triplicates per group. Then each group of cells was digested into single cell suspensions and plated on ultra-low adherent wells of 24-well plates (Corning) at 400 cells per well in sphere formation medium (1:1 DMEM/F12 medium supplemented with 1 × ITS, 1 × NEAA, 1 × B27, 0.2 % BSA, EGF 20 ng/ml and bFGF 20 ng/ml, Invitrogen, USA). Two weeks later, for A549 cells infected with scramble RNA, sh-NLK1, or sh-NLK2, the spheres with diameter over 100 μM were collected by centrifugation (300 g, 5 min) and counted. The spheres from untreated group were digested into single cell suspensions, treated with PBS or 5 mM metformin, and plated on ultra-low adherent 24-well plates at 400 cells per well for second round sphere formation. One week later, the spheres with diameter over 100 μM were collected and counted. Then, spheres were digested into single cell suspensions for CD133+ cell analysis.

### Data analysis

Results are expressed as the mean ± standard deviation (SD). The correlation between immunocytochemical labeling of NLK and other clinical pathology parameters was analyzed by the *χ*^2^ test. One-way ANOVA was used for comparisons of multiple groups. Comparisons between groups for statistical significance were carried out using independent *t* test. A *p* value of <0.05 was considered statistically significant. All statistical analyses were performed by using SPSS version 18.0 software for Windows (SPSS Inc., Chicago, IL, USA). Results are expressed as the mean ± standard deviation.

## Results

### NLK expression is up-regulated in NSCLC tissues

We first examined the expression levels of NLK in 121 NSCLCs and 92 benign lung tissue patient samples. Representative images of NSCLC and benign lung tissue were shown by H&E staining (Fig. 1a, d). NLK-positive staining was confined mainly to the nucleus and cytoplasm (Fig. 1b, c) compared to a negatively stained benign lung tissue (Fig. 1e, f). Table 1 shows the number and percentage of NLK-positive samples for each group. NLK-positive staining was detected in 62 out of 121 (51.2 %) of the samples taken from primary tumors of NSCLC, but only 4 out of 92 (4.4 %) of the benign lung samples (*p* < 0.001). Notably, expression of NLK was correlated with NSCLC Tumor size (T stage) (*p* = 0.016). However, there was no correlation between NLK expression and regional lymph node metastasis (*p* = 0.24), suggesting that NLK expression may be associated with lung cancer cell growth.

**Table 1:** Immunocytochemical staining for NLK in samples from NSCLC and benign pulmonary disease

Characteristic	No. of samples	No. of positive samples (%)	Statistical significance
Normal lung tissues	92	4 (4.4)	*p* = 0.000
Primary tumors of NSCLC	121	62 (51.2)	
Pathology			
Squamous cell carcinoma	61	31 (50.8)	*p* = 0.926
Adenocarcinoma	60	31 (51.7)	
Tumor size (T stage)			
T1/ T2	98	45 (45.0)	*p* = 0.016
T3/ T4	23	17 (73.3)	
Lymph node metastasis			
Positive	60	34 (56.7)	*p* = 0.236
Negative	61	28 (45.9)	

### NLK knockdown inhibits proliferation of NSCLC cells in vitro

**Fig. 2:**
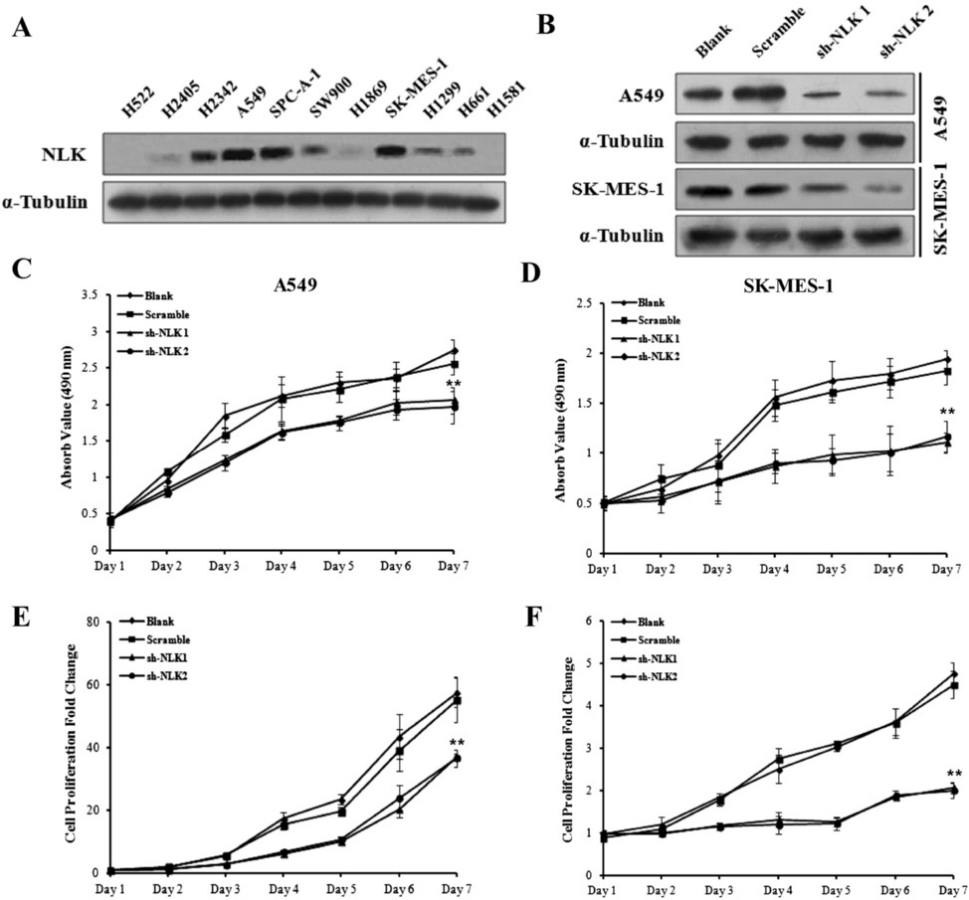
Knockdown of NLK in NSCLC cell lines inhibits cell proliferation. **a** NLK protein levels were assessed by western blot analysis in 11 human NSCLC cell lines. H522, H2405, H1869, and H1581 were negative for NLK expression, whereas H2342, A549, SPC-A-1, SW900, SK-MES-1, H1299, and H661 cells all showed positive expression of NLK at various levels. α-tubulin was used as loading control. **b** Verification of NLK knockdown in A549 and SK-MES-1 cell lines by western blot using lentivirus-mediated shRNA. **c**, **d** MTS assay showing reduced cell growth in A549 and SK-MES-1 cells infected with NLK-shRNA. **p < 0.01. **e**, **f** Cell counting assay showing decreased cell numbers in A549 and SK-MES-1 cells infected with NLK-shRNA. **p < 0.01

The identification of NLK in such a significant number of NSCLC tissue samples prompted us to examine NLK protein levels in multiple human lung cancer cell lines. By western blot analysis, out of 11 human NSCLC cell lines, H522, H2405, H1869, and H1581 were negative for NLK expression, whereas H2342, A549, SPC-A-1, SW900, SK-MES-1, H1299, and H661 cells all showed positive expression of NLK at various levels (Fig. 2a).

To further study the function of NLK in NSCLCs, we choose to knockdown NLK in A549 and SK-MES-1 cells, both of which show high expression of NLK (Fig. 2a). Both cell lines were either untreated or infected with scramble shRNA or two different shRNA against NLK. NLK protein levels were reduced by both NLK-shRNAs in A549 and SK-MES-1 cells, confirming the effectiveness of the shRNAs and the specificity of the anti-NLK antibody (Fig. 2b).

Then we investigated the impact of NLK silencing on cell proliferation; MTS assay and cell growth curve analysis were performed using both A549 and SK-MES-1 cells. MTS assay showed that knockdown of NLK significantly inhibited cell proliferation in A549 and KS-MES-1 cells (*p* < 0.001 for each. Fig. 2c, d). Cell growth curve analysis also showed that depletion of NLK suppressed the growth rate of A549 and SK-MES-1 cells significantly (*p* = 0.004 for A549, *p* < 0.001 for SK-MES-1. Fig. 2e, f) compared to cells infected with scramble shRNA. For MTS assay or cell growth analysis in both cell lines, no significant difference was found between scramble shRNA-infected cells and non-infected cells.

### NLK knockdown induces G1/S phase arrest of NSCLC cell lines

**Fig. 3:**
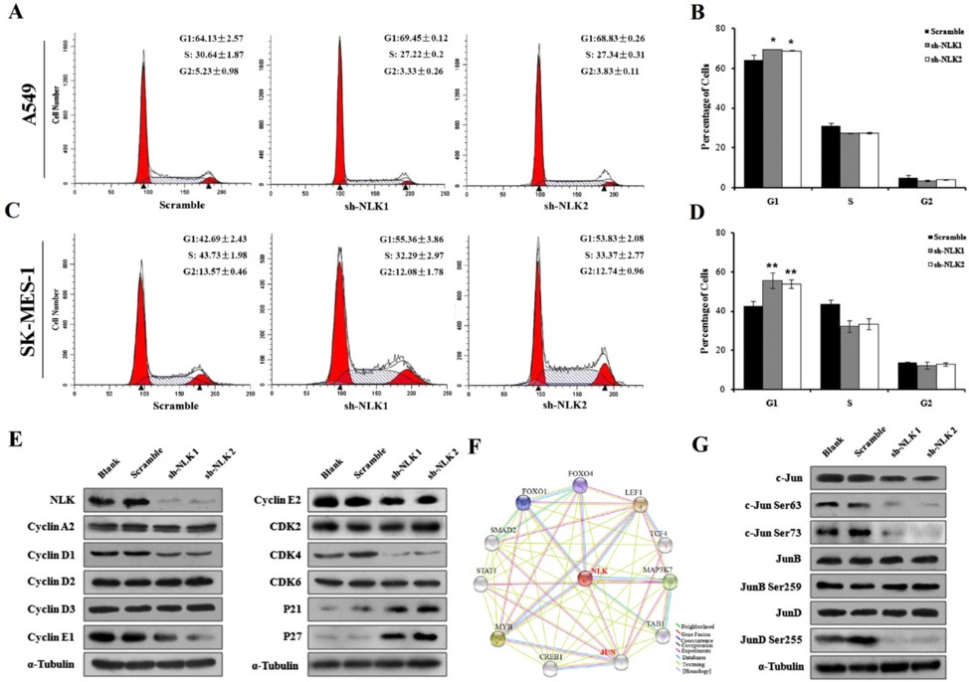
Effects of NLK silencing on cell cycle of NSCLC cell lines. **a**–**d** Flow cytometric analysis and quantitation of cell cycle in A549 (**a**, **b**) and SK-MES-1 cells (**c**, **d**) infected with control or NLK-shRNA. **e** Western blots showing that knockdown of NLK in A549 cells affects the expression of multiple cell cycle-related proteins including Cyclins, CDKs, and CDKIs. α-tubulin was used as loading control. **f** It is predicted that there might exist some correlation between JUN proteins and NLK. **g** Western blots showing that knockdown of NLK in A549 cells affects the expression and phosphorylation of Jun family members including c-Jun, JunB, and JunD. α-tubulin was used as loading control

Next, Flow cytometric analysis was performed to examine the detailed cell cycle distribution of A549 (*p* = 0.009. Fig. 3a, b) and SK-MES-1 (*p* = 0.003. Fig. 3c, d) cells. After NLK silencing, the proportion of cancer cells in the G1 phase was significantly increased in both cell lines, and those in the S phase was significantly decreased concomitantly. These data suggest that NLK mediates the G1/S transition in A549 and SK-MES-1 cells.

To G1/S transition in the cell cycle is controlled by different cyclins, cyclin-dependent kinases (CDKs), and cyclin-dependent kinases inhibitors (CDKIs) [26]. We examined the effect of NLK silencing on a wide range of these molecules, including the expression of Cyclin A2, D1, D2, D3, E1 and E2, CDK2, 4 and 6, p21 and p27. After NLK knockdown in A549 cells, the protein levels of the cell cycle promoters Cyclin D1, E1 and E2, and CDK4 markedly decreased, while those of the cell cycle inhibitors p21 and p27 were dramatically up-regulated (Fig. 3e).

To further investigate the mechanism how NLK regulates those cell cycle promoters and inhibitors, we find out that there might exist some correlation between JUN family proteins and NLK (http://string.embl.de/, Fig. 3f). Meanwhile, activation of c-Jun and JunD are reported to stimulate Cyclin D1 expression and promote cell cycle progress [27, 28]. Consistently, after NLK knockdown, we observed a decrease in c-Jun protein levels as well as phosphorylation on serines 63 and 73, and phosphorylated JunD on serine 255 (Fig. 3g).

Collectively, these data indicated that NLK might play a crucial role in regulating the cell cycle transition in NSCLC via modulation of JUN family proteins which further affect cell cycle regulators, specifically cyclins, CDKs, and CDKIs.

### NLK knockdown reduces tumorigenicity of lung cancer cells in vivo

**Fig. 4:**
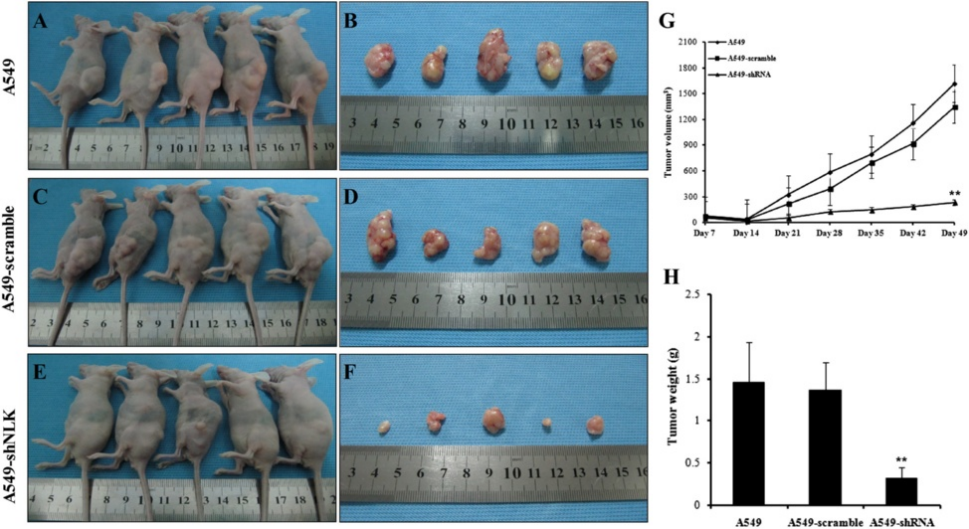
NLK influences tumorigenesis in nude mice model. A549 (**a**, **b**), A549 infected with scramble (**c**, **d**) and A549 infected with shRNA against NLK (**e**, **f**) were implanted subcutaneously into the left flank of nude mice. Representative pictures show mice and tumors from each group at 49 days post-inoculation. **g** Tumor volumes in three groups over time. Tumors were measured on the indicated days using Vernier calipers. **p < 0.01. **h** *Bar graph* showing tumor weights at 49 days post-injection. **p < 0.01

In order to examine the effect of NLK depletion on lung cancer cell tumorigenicity in an in vivo model, we inoculated A549, A549-scramble and A549-shRNA cells subcutaneously into the left flank of female athymic nude mice. Cells mixed with Matrigel were completely absorbed 3 days after subcutaneous inoculation, and palpable tumors could be detected 7 days after injection. shRNA mediated knockdown of NLK in A549 cells resulted in a markedly reduced growth rate (*p* < 0.001) and tumor weight (*p* < 0.001) compared with tumors formed from A549-scramble cells (Fig. 4). These data provide in vivo evidence that NLK contributes significantly to the tumorigenicity of lung cancer cells both in vitro and in vivo.

### Metformin selectively inhibits NLK expression and proliferation in NSCLC cells

**Fig. 5:**
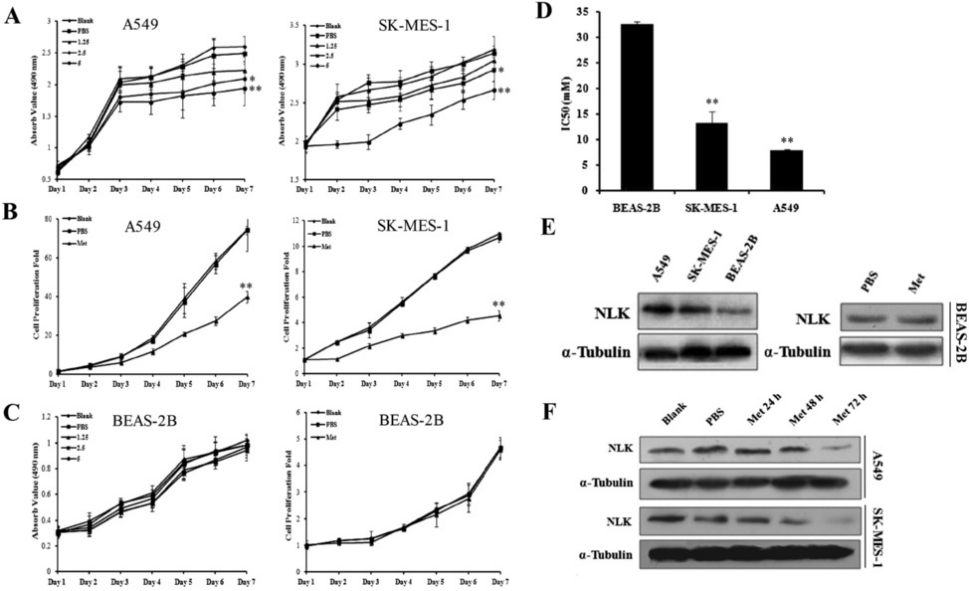
Metformin treatment inhibits cell proliferation in NSCLC cell lines, consistent with down-regulation of NLK protein levels. **a** MTS assay showing reduced cell growth in A549 and SK-MES-1 cells by different dosage of metformin treatment. *p < 0.05, **p < 0.01. **b** Cell counting assay showing decreased cell numbers in A549 and SK-MES-1 cells by the indicated dosages of metformin treatment. **p < 0.01. **c** The growth of normal human bronchial epithelial cells (BEAS-2B) is not affected by metformin treatment shown by MTS and cell counting assay. **d** A549 (IC50 7.97 ± 0.17) and SK-MES-1 (IC50 13.36 ± 0.44) were found to be more sensitive to metformin than BEAS-2B (IC50 32.57 ± 0.64). **p < 0.01. **e** Western blots showing that NLK expression in BEAS-2B is lower than A549 and SK-MES-1 cells and the treatment of metformin did not decrease NLK protein expression level markedly. **f** Western blots showing decreased NLK protein level upon metformin treatment (5 mM) with indicated times in A549 and SK-MES-1 cells. α-tubulin was used as loading control

**Fig. 6:**
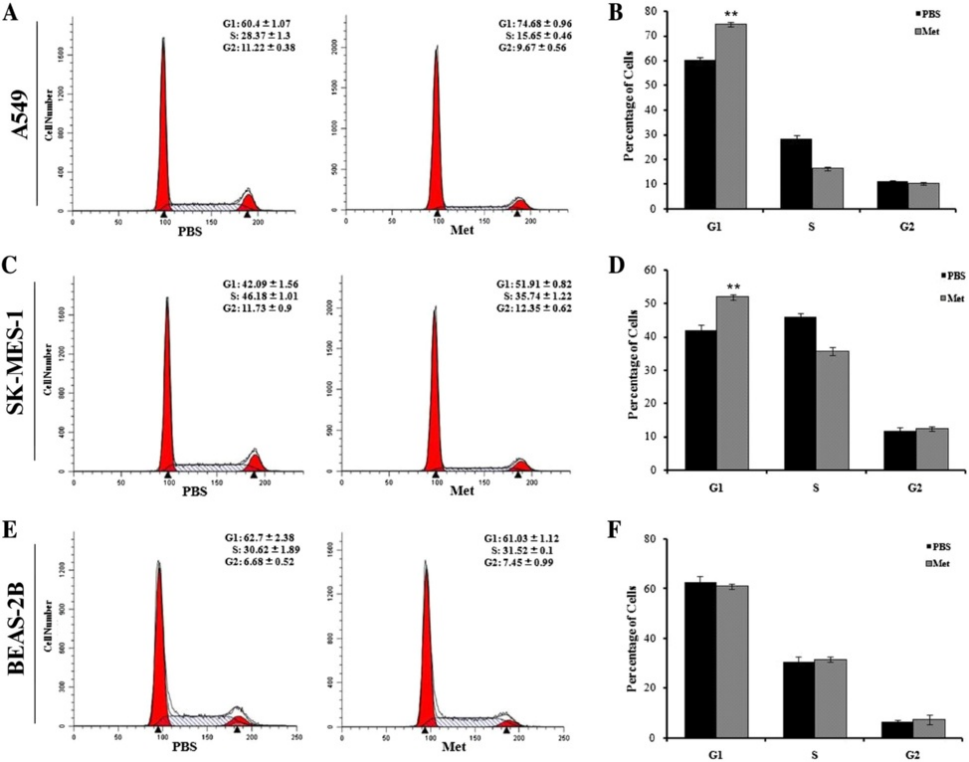
Effects of metformin treatment on cell cycles of NSCLC and immortalized noncancerous human bronchial epithelial cells. Cell cycle of PBS and metformin-treated A549 (**a**), SK-MES-1 (**c**), and BEAS-2B (**e**) cells were measured by flow cytometric analysis. *Bar graph* showing quantification results of A549 (**b**), SK-MES-1 (**d**), and BEAS-2B (**f**) cells in different phases of cell cycle. **p < 0.01

**Fig. 7:**
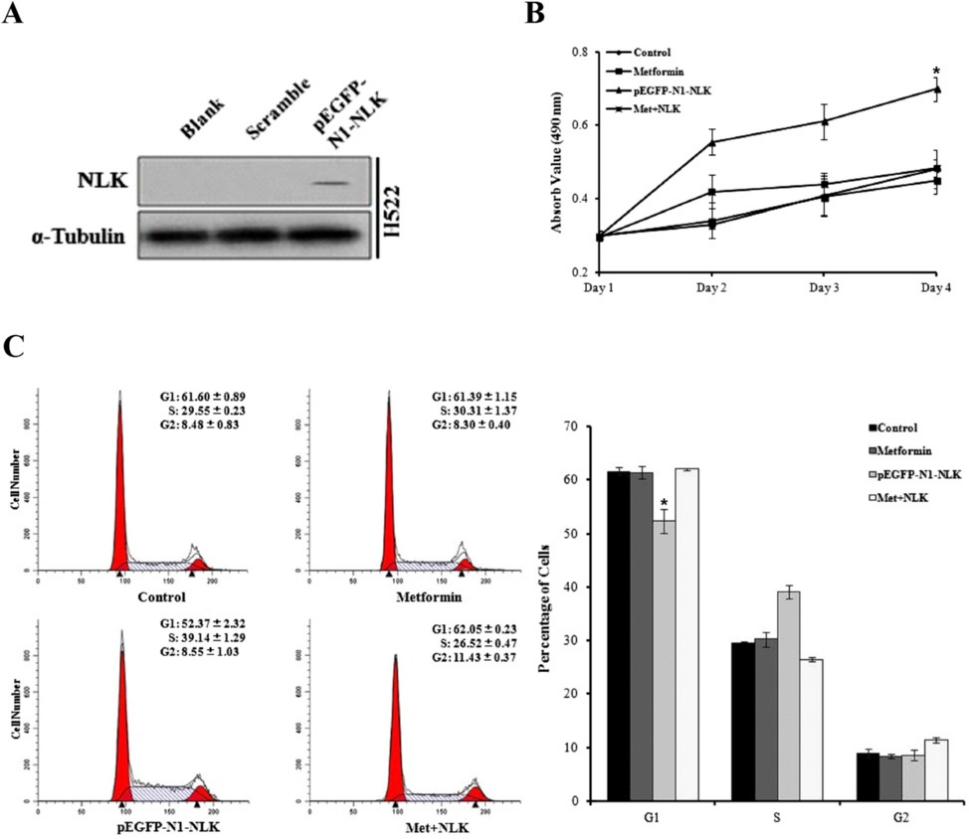
Ectopic expression of NLK increased cell proliferation ability and metformin compromised the promoting activity of NLK. **a** Expression of NLK in H522 lung cancer cell line after pEGFP-N1-NLK plasmids transfection. **b**–**c** MTS assay and cell cycle analysis showing increased cell growth in H522 cells transfected with NLK expression plasmids, and metformin treatment reversed the pro-proliferation activity of NLK. An empty vector served as control. All transfected cells were collected for the next experiments 48 h after transfection. *p < 0.05

Studies on the novel role of the diabetes drug metformin in cancer therapy are emerging, but the molecular mechanisms remain not fully elucidated. When the NSCLC cell lines A549 and SK-MES-1 were subjected to metformin treatment, their proliferative activities were inhibited by metformin in a dose-dependent manner, as demonstrated by assays of MTS and cell counting (*p* = 0.003 for A549, *p* < 0.001 for SK-MES-1. Fig. 5a, b). Interestingly, this growth inhibition was not observed in the normal human bronchial epithelial cells BEAS-2B (Fig. 5c). As compared with the IC50 results (IC50 = 32.57, 7.97 and 13.36 mM for BEAS-2B, A549 and SK-MES-1, respectively), BEAS-2B showed less sensitive to metformin treatment (*p* < 0.001. Fig. 5d). Moreover, we proved that NLK expression is lower in BEAS-2B than NSCLC cells and not affected by metformin treatment (Fig. 5e). On the contrary, metformin decreased NLK protein levels in A549 and SK-MES-1 cells along with time (Fig. 5f). Consistent with the NLK knockdown effect, metformin treatment significantly arrested more NSCLC cells (*p* < 0.001 for each group. Fig. 6a–d) but not normal epithelial cells (Fig. 6e, f) in the G1 phase of the cell cycle. To explore the effects of NLK in the anti-cancer activities of metformin, we constructed plasmids expressing NLK. Transient transfection of the plasmids led to ectopic expression of NLK in H522 cells, an NLK protein negative expression cell line measured by Western Blot analysis (Figs. 2a and 7a). In pEGFP-N1-NLK plasmids transfected H522 cells, NLK overproduction increased cell proliferation ability, and metformin treatment compromised the pro-proliferation activity of NLK (Fig. 7b, c). Collectively, these data suggest that NLK is involved in metformin mediated inhibition of NSCLC growth.

### NLK silencing and metformin repress cancer stemness of A549 cells

**Fig. 8:**
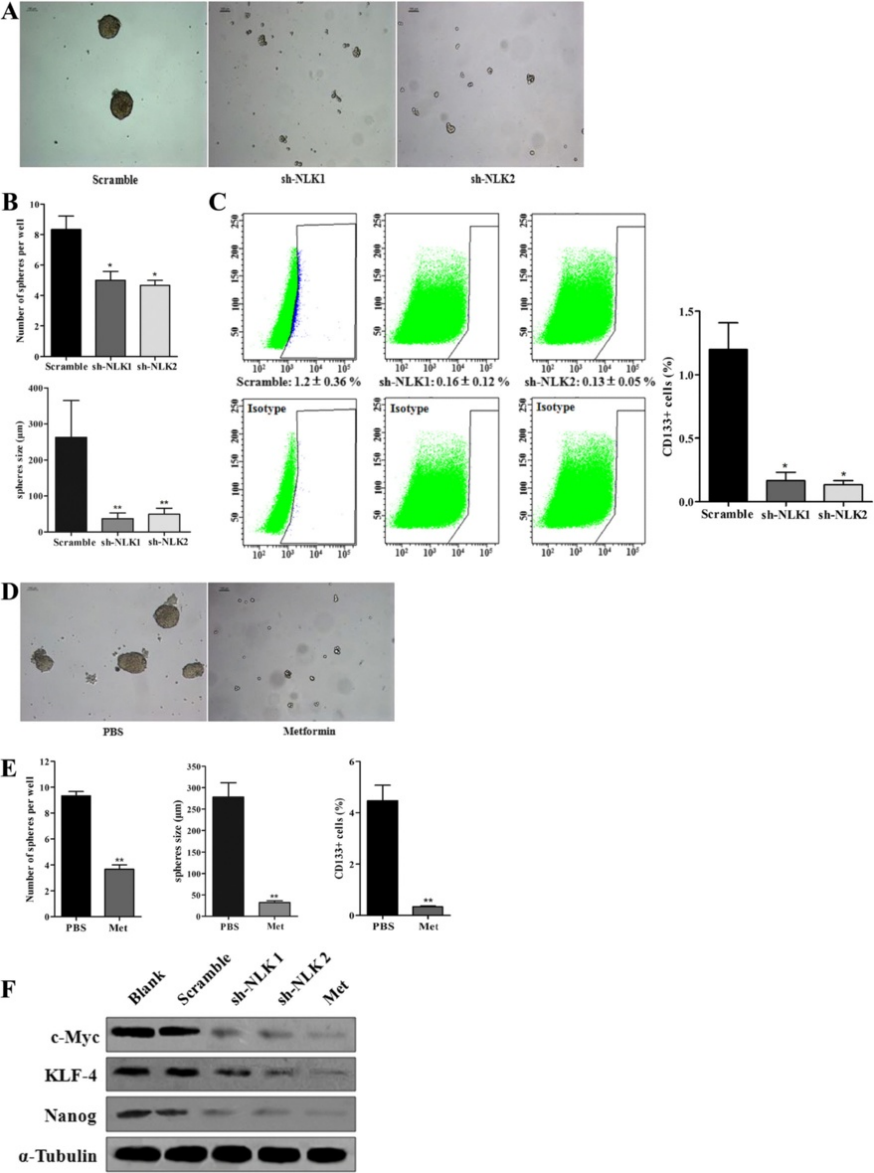
**a** NLK knockdown and metformin treatment reduce A549 cell stemness. Tumorsphere formation assay was performed in A549 infected with either scramble or NLK-shRNA. **b** *Bar graph* showing quantification results of numbers and diameter of spheres formed per well of each group. **c** Flow cytometric analysis of cell surface marker CD133 expression in A549 cells infected with scramble or NLK-shRNA. *Bar graph* showing quantification results of percentage of CD133+ cells. **d** Tumorsphere formation assay was performed in A549 treated with PBS or metformin. **e** *Bar graph* showing quantification results of numbers and diameter of spheres formed per well and quantification results of CD133+ cells percentage. **f** Both NLK knockdown and metformin treatment significantly decreased the expressions of Nanog, c-Myc and KLF4 in A549 cells

Since cancer stem cells (CSCs) play an important role in maintaining cancer cell populations, targeting specific components of CSCs regulators might open up a new strategy for cancer treatment. In our study, NLK knockdown significantly inhibited the tumor sphere formation from A549 cells (*p* = 0.012, *p* < 0.001 for each. Fig. 8a, b), where the expression of cancer stem cell marker CD133 is dramatically reduced (*p* = 0.002. Fig. 8c). Moreover, metformin treatment showed the same effect on CSCs properties, (*p* = 0.002, *p* < 0.001, *p* = 0.007 for each. Fig. 8d, e). Although it is considered that CD133 is one of key biomarkers for isolation and characterization of stem cells [29], it is confirmed that CD133 expression is not restricted to cancer initiating cells in the human lung cancer cells [30]. Thus, we measured stem cell-associated markers including Nanog, c-Myc, and KLF4. Accordingly, we found that both NLK knockdown and metformin treatment decreased the expressions of Nanog, c-Myc, and KLF4 significantly (Fig. 8f). Taken together, our findings indicate that cancer stem cell stemness is significantly decreased by NLK silencing or metformin treatment.

## Discussion

Tumorigenesis is characterized by uncontrolled cell cycle progression, associated with aberrant alterations of genes or proteins related to regulation of cell proliferation [31]. Thus, identification of genes and their products involved in cell growth modulation is critical in developing effective strategies for cancer therapy.

In this study, we showed by IHC that NLK expression was up-regulated in NSCLC tissues compared with benign tissues (*p* < 0.001), and correlated with NSCLC T stage (*p* < 0.05). Silencing NLK with shRNA reduced the proliferation and tumorigenicity of NSCLC cell lines both in vitro and in vivo, suggesting a critical role for NLK in maintaining of the malignant NSCLC phenotype. NLK controlled G1/S cell cycle progression by modulating the expression of Cyclin D1, E1 and E2, CDK4, p21 and p27. Activation of JUN family proteins can promote cell cycle progression through induction of cell cycle promoters like cyclins and CDKs and repression of cell cycle inhibitors like CDKIs. Our data show that the expression of c-Jun and activity of c-Jun and JunD are greatly reduced by NLK knockdown, which explains the down-regulation of Cyclin D1, E1 and E2, CDK4 and up-regulation of p21 and p27 after NLK knockdown. All of these changes in cyclins, CDKs and CDKIs are consistent with cell cycle arrest by NLK knockdown. Although NLK is a crucial factor for NSCLC tumorigenicity, some NSCLC cell lines showed negative NLK expression. We suspect that these cell lines are originally from different populations with various genetic backgrounds and pathogenic factors.

Our results are supported by recent reports that NLK expression is significantly up-regulated in human hepatocellular and gallbladder carcinoma cells and that targeted disruption of NLK results in suppression of cell growth and cell cycle transition arrest [13, 32]. However, contrasting studies demonstrate that NLK expression is lower in tumors compared with normal tissues, and moreover, that induction of NLK can induce apoptosis of tumor cells [12, 14, 33].

This discrepancy in results may be attributed in part to the ability of NLK to activate a variety of different signaling pathways. Numerous studies show that non-canonical Wnt-5a/Ca^2+^ [34], TGF-β [35], and p38 MAPK [36] signal transduction pathways are partially dependent on the activation of NLK. Activated NLK can bind directly to and phosphorylate transcription factors, regulating their transcriptional activity or inducing degradation in a proteasome-dependent manner [10, 37–42]. In addition, NLK can phosphorylate the C-terminal domain of CREB binding protein (CBP)/p300, and may therefore also suppress a wide range of transcription factors in an indirect manner [11].

A further level of complexity may lie in the upstream regulation of NLK itself. A recent study shows that p38 MAPK directly interacts with NLK to regulate its kinase activity [36] while several studies indicate that over-expression of NLK alone is sufficient for activation of its kinase activity, suggesting that over-expressed NLK can be active without the requirement of upstream kinases [43, 44]. We propose that in different tissue-derived carcinomas, NLK may be differentially activated and may act through diverse pathways to influence the expression of various target genes resulting in distinct biological behaviors.

Since the recognition of EGFR as a therapeutic target, EGFR tyrosine kinase inhibitors (TKIs) have been widely used in treating NSCLC patients, which has been a major breakthrough for lung cancer treatment. Unfortunately, most patients who initially responded to EGFR-TKIs would eventually develop acquired resistance. Many small molecule agents, long non-coding RNAs (lncRNA) and monoclonal antibodies (McAb) targeting signaling pathways are potential therapeutic regimens for overcoming resistance such as Dacomitinib, temsirolimus, everolimus, and GAS which were potential treatment agents for lung cancer [45–47]. Moreover, it is proved that metformin could overcome TKI resistance and inhibit lung cancer cell proliferation [48]. However, the biological role of metformin and its mechanism in lung cancer proliferation remain largely unknown.

Intriguingly, in our study metformin treatment reduces NLK expression, arrests the cell cycle arrest and potentiates anti-proliferation selectively in cancer cells but not the normal lung epithelial cells. Furthermore, our results clearly show that tumor sphere formation ability, CD133+ cancer cell population, and the expression level of stem cell markers (Nanog, c-Myc and TLF4) were significantly reduced by both NLK knockdown and metformin treatment, indicating NLK may be responsible for the inhibition of cancer cell self-renewal and stemness achieved by metformin.

In summary, we have shown that NLK expression is significantly up-regulated in NSCLC. Moreover, abnormal expression of NLK is associated with the malignant phenotype of NSCLC. In addition, we have also demonstrated that knockdown of NLK significantly inhibits cell proliferation and tumorigenecity might through inhibition of JUN family proteins. Meanwhile, cancer cell self-renewal and stemness were inhibited by NLK knockdown. Metformin treatment decreased NLK expression and recapitulated the phenotype observed by NLK knockdown, indicating that NLK may be a contributing factor and underlying mechanism for the inhibition of growth and stemness observed in lung cancer cells. Therefore, targeting NLK could be a potential therapeutic strategy for NSCLC, and metformin treatment may be an approach to pursue.

## References

[1] World Health Organization. Cancer: fact sheet no. 297. World Health Organization website. 2011. http://www.who.int/mediacentre/factsheets/fs297/en. Accessed September 10, 2011.

[2] Jemal A, Siegel R, Ward E, Murray T, Xu J, Smigal C (2006). Cancer statistics, 2006. CA Cancer J Clin.

[3] Edelman AM, Blumenthal DK, Krebs EG (1987). Protein serine/threonine kinases. Annu Rev Biochem.

[4] Hynes NE (2000). Tyrosine kinase signalling in breast cancer. Breast Cancer Res.

[5] Hynes NE, Boulay A (2006). The mTOR pathway in breast cancer. J Mammary Gland Biol Neoplasia.

[6] Brott BK, Pinsky BA, Erikson RL (1998). Nlk is a murine protein kinase related to Erk/MAP kinases and localized in the nucleus. Proc Natl Acad Sci U S A.

[7] Verheyen EM, Mirkovic I, MacLean SJ, Langmann C, Andrews BC, MacKinnon C (2001). The tissue polarity gene nemo carries out multiple roles in patterning during Drosophila development. Mech Dev.

[8] Mirkovic I, Charish K, Gorski SM, McKnight K, Verheyen EM (2002). Drosophila nemo is an essential gene involved in the regulation of programmed cell death. Mech Dev.

[9] Choi KW, Benzer S (1994). Rotation of photoreceptor clusters in the developing Drosophila eye requires the nemo gene. Cell.

[10] Ishitani T, Ninomiya-Tsuji J, Nagai S, Nishita M, Meneghini M, Barker N (1999). The TAK1-NLK-MAPK-related pathway antagonizes signalling between beta-catenin and transcription factor TCF. Nature.

[11] Yasuda J, Yokoo H, Yamada T, Kitabayashi I, Sekiya T, Ichikawa H (2004). Nemo-like kinase suppresses a wide range of transcription factors, including nuclear factor-kappaB. Cancer Sci.

[12] Yasuda J, Tsuchiya A, Yamada T, Sakamoto M, Sekiya T, Hirohashi S (2003). Nemo-like kinase induces apoptosis in DLD-1 human colon cancer cells. Biochem Biophys Res Commun.

[13] Jung KH, Kim JK, Noh JH, Eun JW, Bae HJ, Xie HJ (2010). Targeted disruption of Nemo-like kinase inhibits tumor cell growth by simultaneous suppression of cyclin D1 and CDK2 in human hepatocellular carcinoma. J Cell Biochem.

[14] Emami KH, Brown LG, Pitts TE, Sun X, Vessella RL, Corey E (2009). Nemo-like kinase induces apoptosis and inhibits androgen receptor signaling in prostate cancer cells. Prostate.

[15] Evans JM, Donnelly LA, Emslie-Smith AM, Alessi DR, Morris AD (2005). Metformin and reduced risk of cancer in diabetic patients. BMJ.

[16] Anisimov VN (2015). Metformin for prevention and treatment of colon cancer: a reappraisal of experimental and clinical data. Curr Drug Targets.

[17] Moyad MA, Vogelzang NJ (2015). Heart healthy equals prostate healthy and statins, aspirin, and/or metformin (S.A.M.) are the ideal recommendations for prostate cancer prevention. Asian J Androl.

[18] Yue W, Yang CS, DiPaola RS, Tan XL (2014). Repurposing of metformin and aspirin by targeting AMPK-mTOR and inflammation for pancreatic cancer prevention and treatment. Cancer Prev Res.

[19] Edge SB, Compton CC (2010). The American Joint Committee on Cancer: the 7th edition of the AJCC cancer staging manual and the future of TNM. Ann Surg Oncol.

[20] Li M, Zhang S, Wang Z, Zhang B, Wu X, Weng H (2013). Prognostic significance of nemo-like kinase (NLK) expression in patients with gallbladder cancer. Tumour Biol.

[21] Shi Z, Liang YJ, Chen ZS, Wang XW, Wang XH, Ding Y (2006). Reversal of MDR1/P-glycoprotein-mediated multidrug resistance by vector-based RNA interference in vitro and in vivo. Cancer Biol Ther.

[22] Kleinman HK, McGarvey ML, Hassell JR, Star VL, Cannon FB, Laurie GW (1986). Basement membrane complexes with biological activity. Biochem.

[23] Fridman R, Kibbey MC, Royce LS, Zain M, Sweeney M, Jicha DL (1991). Enhanced tumor growth of both primary and established human and murine tumor cells in athymic mice after coinjection with Matrigel. J Natl Cancer Inst.

[24] Wolf M (2008). Influence of matrigel on biodistribution studies in cancer research. Pharmazie.

[25] Bao B, Wang Z, Ali S, Ahmad A, Azmi AS, Sarkar SH (2012). Metformin inhibits cell proliferation, migration and invasion by attenuating CSC function mediated by deregulating miRNAs in pancreatic cancer cells. Cancer Prev Res.

[26] Grana X, Reddy EP (1995). Cell cycle control in mammalian cells: role of cyclins, cyclin dependent kinases (CDKs), growth suppressor genes and cyclin-dependent kinase inhibitors (CKIs). Oncogene.

[27] Toualbi-Abed K, Daniel F, Guller MC, Legrand A, Mauriz JL, Mauviel A (2008). Jun D cooperates with p65 to activate the proximal kappaB site of the cyclin D1 promoter: role of PI3K/PDK-1. Carcinog.

[28] Schwabe RF, Bradham CA, Uehara T, Hatano E, Bennett BL, Schoonhoven R (2003). c-Jun-N-terminal kinase drives cyclin D1 expression and proliferation during liver regeneration. Hepatol.

[29] Li Z (2013). CD133: a stem cell biomarker and beyond. Exp Hematol Oncol.

[30] Wang P, Suo Z, Wang M, Hoifodt HK, Fodstad O, Gaudernack G (2013). In vitro and in vivo properties of CD133 expressing cells from human lung cancer cell lines. Exp Hematol Oncol.

[31] Doerfler W, Hohlweg U, Muller K, Remus R, Heller H, Hertz J (2001). Foreign DNA integration—perturbations of the genome--oncogenesis. Ann N Y Acad Sci.

[32] Tan Z, Li M, Wu W, Zhang L, Ding Q, Wu X (2012). NLK is a key regulator of proliferation and migration in gallbladder carcinoma cells. Mol Cell Biochem.

[33] Zhang Y, Peng C, Wu G, Wang Y, Liu R, Yang S (2011). Expression of NLK and its potential effect in ovarian cancer chemotherapy. Int J Gynecol Cancer.

[34] Ishitani T, Kishida S, Hyodo-Miura J, Ueno N, Yasuda J, Waterman M (2003). The TAK1-NLK mitogen-activated protein kinase cascade functions in the Wnt-5a/Ca(2+) pathway to antagonize Wnt/beta-catenin signaling. Mol Cell Biol.

[35] Kojima H, Sasaki T, Ishitani T, Iemura S, Zhao H, Kaneko S (2005). STAT3 regulates Nemo-like kinase by mediating its interaction with IL-6-stimulated TGFbeta-activated kinase 1 for STAT3 Ser-727 phosphorylation. Proc Natl Acad Sci U S A.

[36] Ohnishi E, Goto T, Sato A, Kim MS, Iemura S, Ishitani T (2010). Nemo-like kinase, an essential effector of anterior formation, functions downstream of p38 mitogen-activated protein kinase. Mol Cell Biol.

[37] Kanei-Ishii C, Ninomiya-Tsuji J, Tanikawa J, Nomura T, Ishitani T, Kishida S (2004). Wnt-1 signal induces phosphorylation and degradation of c-Myb protein via TAK1, HIPK2, and NLK. Genes Dev.

[38] Kurahashi T, Nomura T, Kanei-Ishii C, Shinkai Y, Ishii S (2005). The Wnt-NLK signaling pathway inhibits A-Myb activity by inhibiting the association with coactivator CBP and methylating histone H3. Mol Biol Cell.

[39] Shi Y, Ye K, Wu H, Sun Y, Shi H, Huo K (2010). Human SMAD4 is phosphorylated at Thr9 and Ser138 by interacting with NLK. Mol Cell Biochem.

[40] Kim S, Kim Y, Lee J, Chung J (2010). Regulation of FOXO1 by TAK1-Nemo-like kinase pathway. J Biol Chem.

[41] Yamada M, Ohnishi J, Ohkawara B, Iemura S, Satoh K, Hyodo-Miura J (2006). NARF, an nemo-like kinase (NLK)-associated ring finger protein regulates the ubiquitylation and degradation of T cell factor/lymphoid enhancer factor (TCF/LEF). J Biol Chem.

[42] Ohkawara B, Shirakabe K, Hyodo-Miura J, Matsuo R, Ueno N, Matsumoto K (2004). Role of the TAK1-NLK-STAT3 pathway in TGF-beta-mediated mesoderm induction. Genes Dev.

[43] Ishitani T, Ishitani S, Matsumoto K, Itoh M (2009). Nemo-like kinase is involved in NGF-induced neurite outgrowth via phosphorylating MAP1B and paxillin. J Neurochem.

[44] Ishitani T, Hirao T, Suzuki M, Isoda M, Ishitani S, Harigaya K (2010). Nemo-like kinase suppresses Notch signalling by interfering with formation of the Notch active transcriptional complex. Nat Cell Biol.

[45] Niu FY, Wu YL (2014). Novel agents and strategies for overcoming EGFR TKIs resistance. Exp Hematol Oncol.

[46] Loewen G, Jayawickramarajah J, Zhuo Y, Shan B (2014). Functions of lncRNA HOTAIR in lung cancer. J Hematol Oncol.

[47] Dong S, Qu X, Li W, Zhong X, Li P, Yang S (2015). The long non-coding RNA, GAS5, enhances gefitinib-induced cell death in innate EGFR tyrosine kinase inhibitor-resistant lung adenocarcinoma cells with wide-type EGFR via downregulation of the IGF-1R expression. J Hematol Oncol.

[48] Li L, Han R, Xiao H, Lin C, Wang Y, Liu H (2014). Metformin sensitizes EGFR-TKI-resistant human lung cancer cells in vitro and in vivo through inhibition of IL-6 signaling and EMT reversal. Clin Cancer Res.

