# Erratum to: NLK functions to maintain proliferation and stemness of NSCLC and is a target of metformin

**DOI:** 10.1186/s13045-016-0239-4

**Published:** 2016-02-17

**Authors:** Dong Suwei, Zeng Liang, Liu Zhimin, Li Ruilei, Zou Yingying, Li Zhen, Ge Chunlei, Lai Zhangchao, Xue Yuanbo, Yang Jinyan, Li Gaofeng, Song Xin

**Affiliations:** Cancer Research Institute of Southern Medical University, Guangzhou, Guangdong People’s Republic of China; Department of Cancer Biotherapy Center, The Third Affiliated Hospital of Kunming Medical University (Tumor Hospital of Yunnan Province), Kunming, Yunnan People’s Republic of China; Department of Pathology, Hunan Tumor Hospital, Changsha, Hunan People’s Republic of China; Department of Pathology and Pathophysiology, Kunming Medical University, Kunming, Yunnan People’s Republic of China; Department of Thoracic Surgery, The Third Affiliated Hospital of Kunming Medical University (Tumor Hospital of Yunnan Province), Kunming, Yunnan, People’s Republic of China

## Erratum

Following the publication of this article [1], the authors noticed that the text contained an error. Within the text ‘TLF4’ should be ‘KLF4’.

## References

[1] Song X (2015). NLK functions to maintain proliferation and stemness of NSCLC and is a target of metformin. J Hematol Oncol.

